# Supplementary material for: Gold Nanoparticles-Functionalized Cotton as Promising Flexible and Green Substrate for Impedometric VOC Detection
Source: Materials (Basel). 2023 Aug 25;16(17):5826. doi: 10.3390/ma16175826 (PMC10488880; doi:10.3390/ma16175826)
Supplement: Supplementary file 1 [file materials-16-05826-s001.zip › materials-2535361-supplementary.pdf]

*materials*

MDPI

*Supplementary Information*

# Gold Nanoparticles-Functionalized Cotton as Promising Flexible and Green Substrate for Impedometric VOC Detection

**Silvia Casalinuovo [1], Daniela Caschera [2,*], Simone Quaranta [2], Virgilio Genova [3], Alessio Buzzin [1], Fulvio Federici [2], Giampiero de Cesare [1], Donatella Puglisi [4] and Domenico Caputo [1]**

1 Department of Information Engineering, Electronics and Telecommunications, Sapienza University of Rome, via Eudossiana 18, **00184** Rome, Italy; silvia.casalinuovo@uniroma1.it (S.C.); alessio.buzzin@uniroma1.it (A.B.); giampiero.decesare@uniroma1.it (G.d.C.); domenico.caputo@uniroma1.it (D.C.)

2 Institute for the Study of Nanostructured Materials CNR-ISMN, Strada Provinciale 35d/9 00010, Montelibretti, 00010 Rome, Italy; daniela.caschera@cnr.it (D.C.); simone.quaranta@cnr.it (S.Q.); fulvio.federici@cnr.it (F.F.)

3 Department of Chemical Engineering, Materials and Environment, Sapienza University of Rome, via Eudossiana 18, **00184** Rome, Italy; virgilio.genova@uniroma1.it (V.G.)

4 Division of Sensor and Actuator Systems, Department of Physics, Chemistry and Biology (IFM), Linköping University, Campus Valla, 58183 Linköping, Sweden; donatella.puglisi@liu.se (D.P.)

* Correspondence: daniela.caschera@cnr.it.

**Citation:** Casalinuovo, S.; Caschera, D.; Quaranta, S.; Genova, V.; Buzzin, A.; Federici, F.; de Cesare, G.; Puglisi, D.; Caputo, D. Gold Nanoparticle - Functionalized Cotton as Promising Flexible and Green Substrate for Impedometric VOC Detection. *Materials* **2023**, *16*, x. https://doi.org/10.3390/xxxxx

Academic Editor: Yuyan Jiang

Received: 16 July 2023
Revised: 7 August 2023
Accepted: 19 August 2023
Published: date

**Copyright:** © 2023 by the authors. Submitted for possible open access publication under the terms and conditions of the Creative Commons Attribution (CC BY) license (https://creativecommons.org/licenses/by/4.0/).

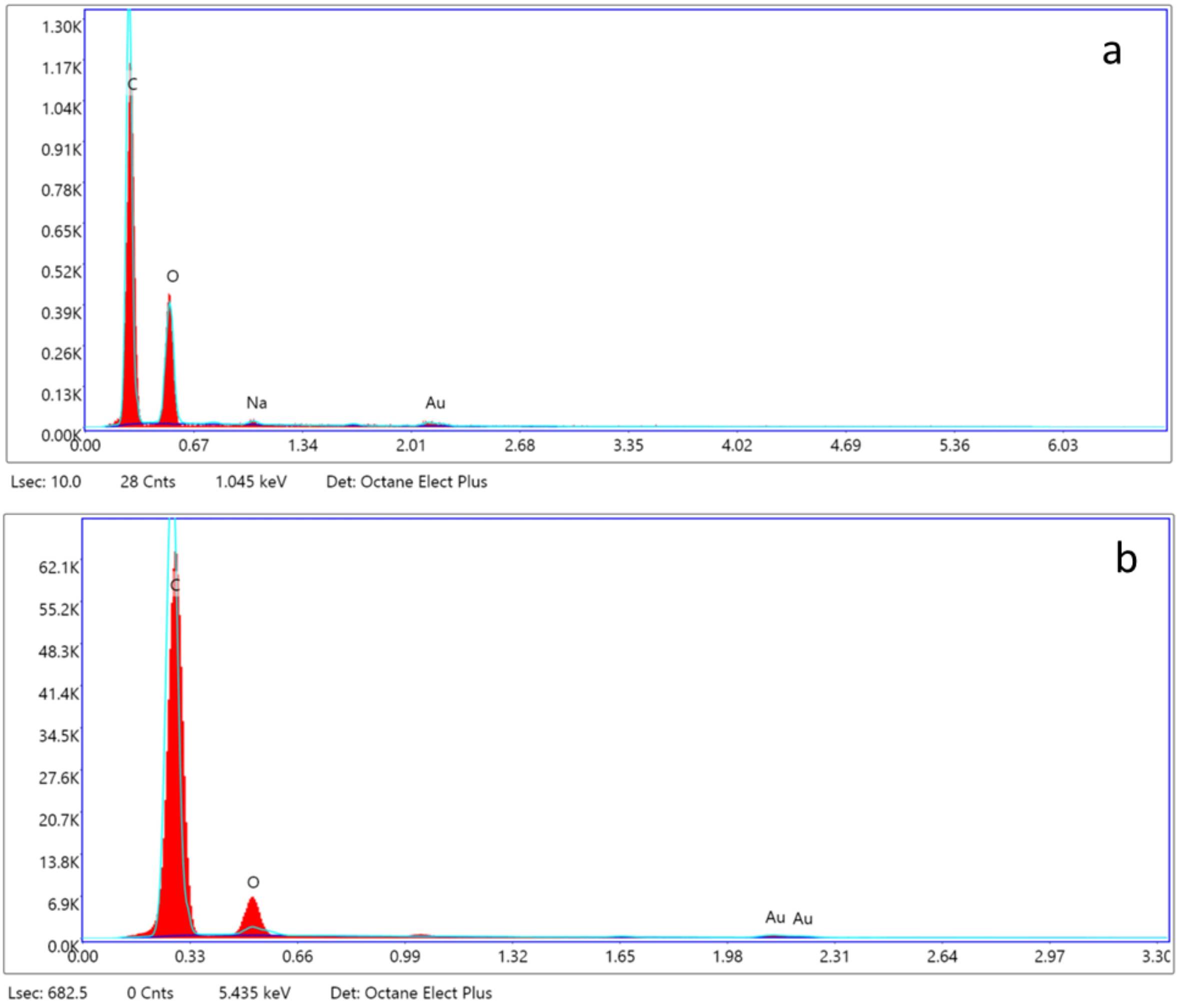


**Figure S1.** EDX spectra for (**a**) AuNP_Citr/COT and (**b**) AuNP_PVP/COT.

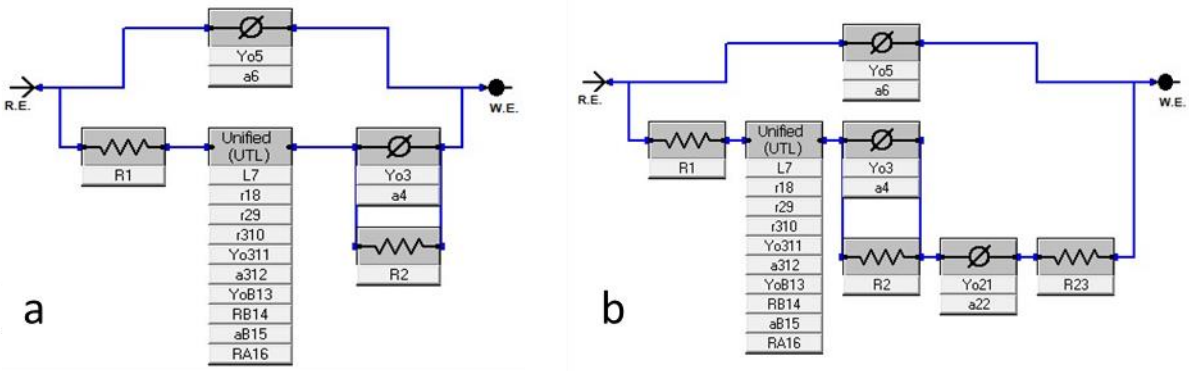


**Figure S2.** Equivalent circuit models of (**a**) AuNP_Citr/COT and AuNP_PVP/COT before and (**b**) after spraying the interested analytes.

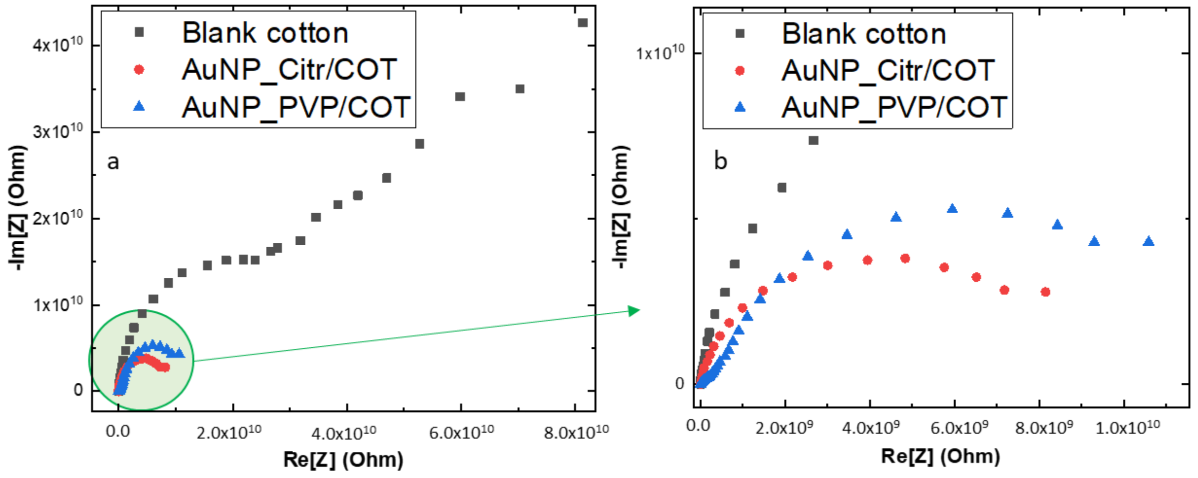


**Figure S3.** (**a**) comparison between the EIS measurements over blank cotton, AuNP_PVP/COT and AuNP_Citr/COT, (**b**) zoom plot at high frequencies.

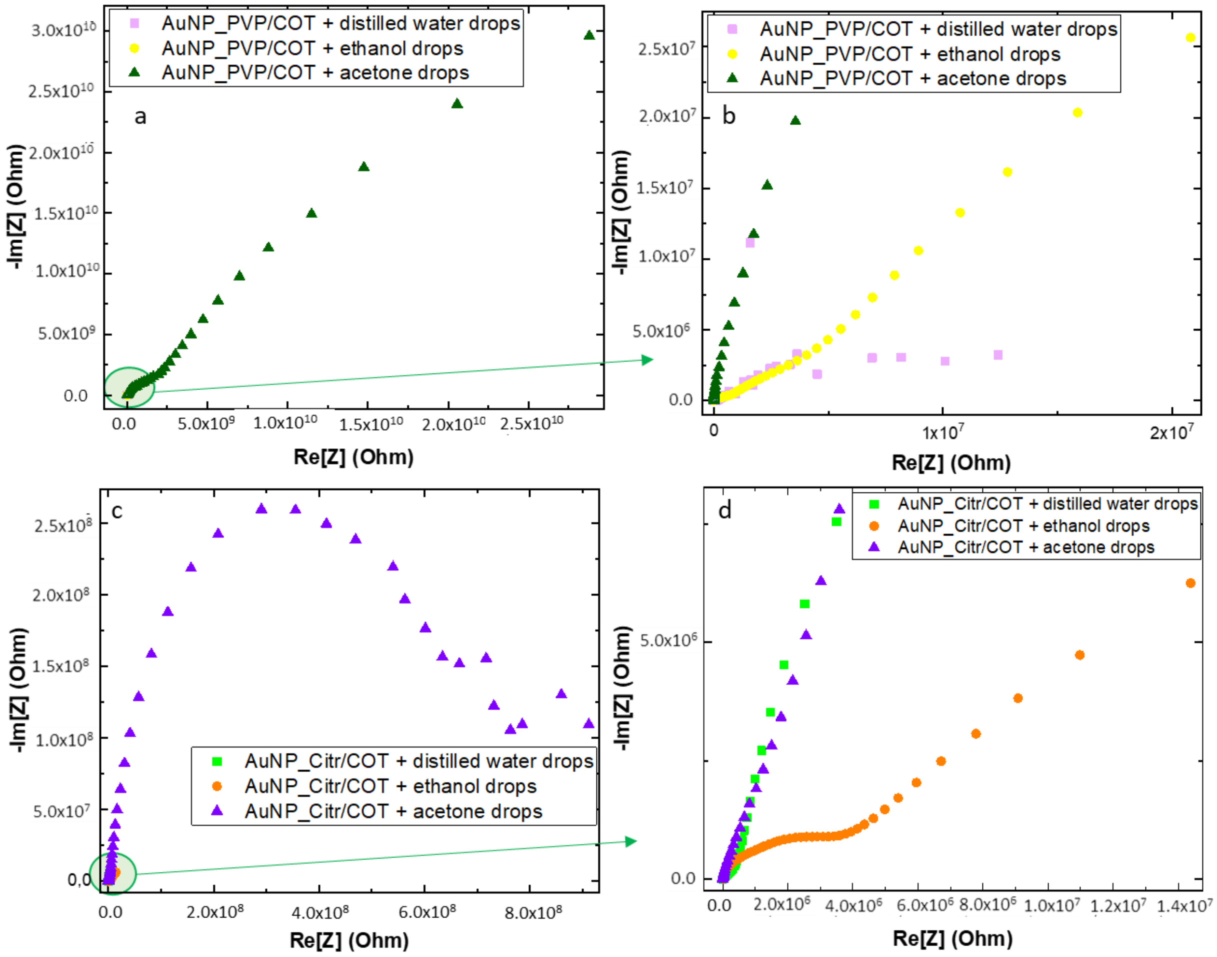


**Figure S4.** (**a,c**) comparison between the EIS measurements over AuNP_PVP/COT and AuNP_Citr/COT after spraying water/ethanol/acetone drops, (**b,d**) zoom plot at high frequencies.

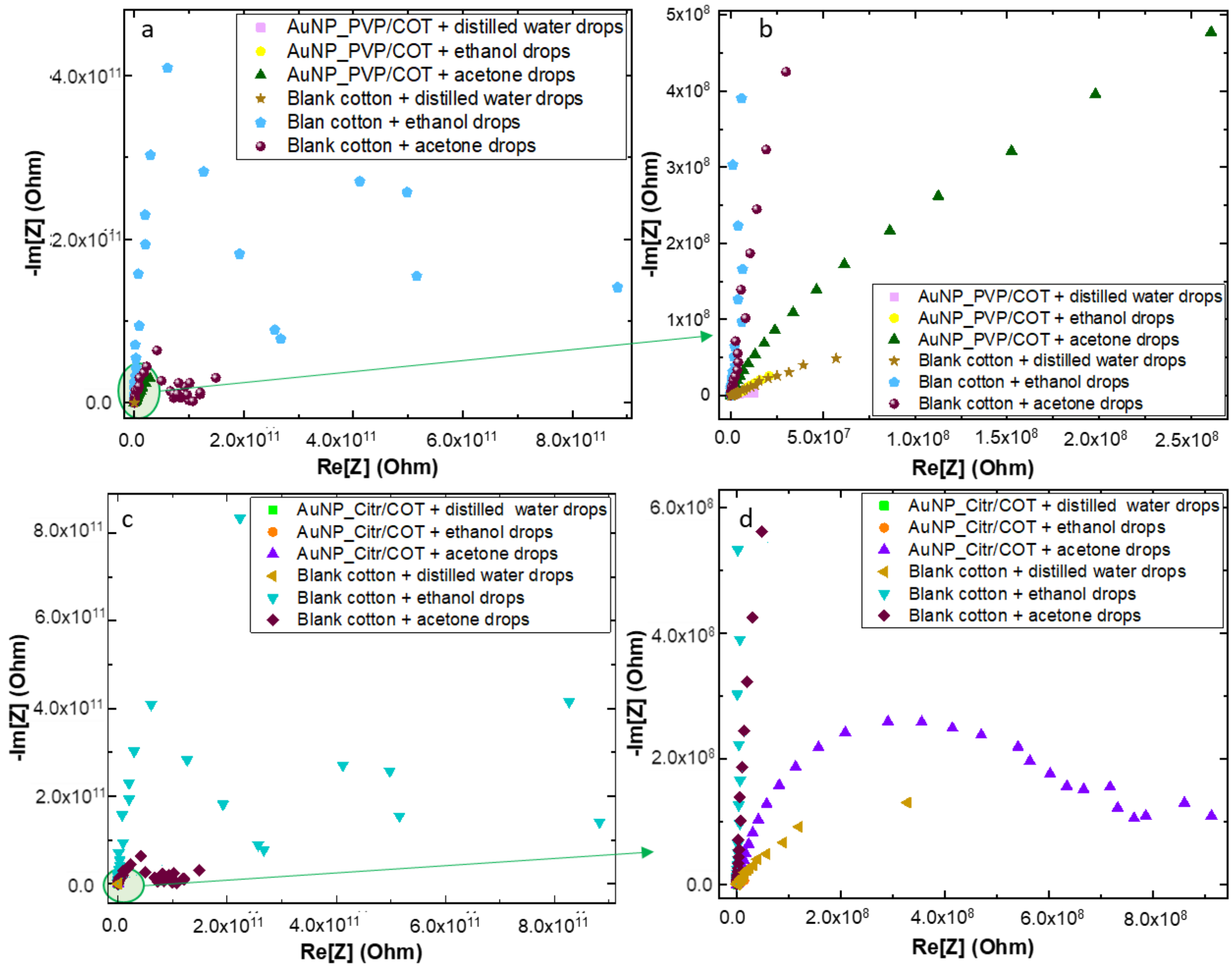


**Figure S5.** (**a**,**c**) comparison between the EIS measurements over the functionalized and pristine fabrics with water/ethanol/acetone drops, (**b**,**d**) zoom plot at high frequencies.

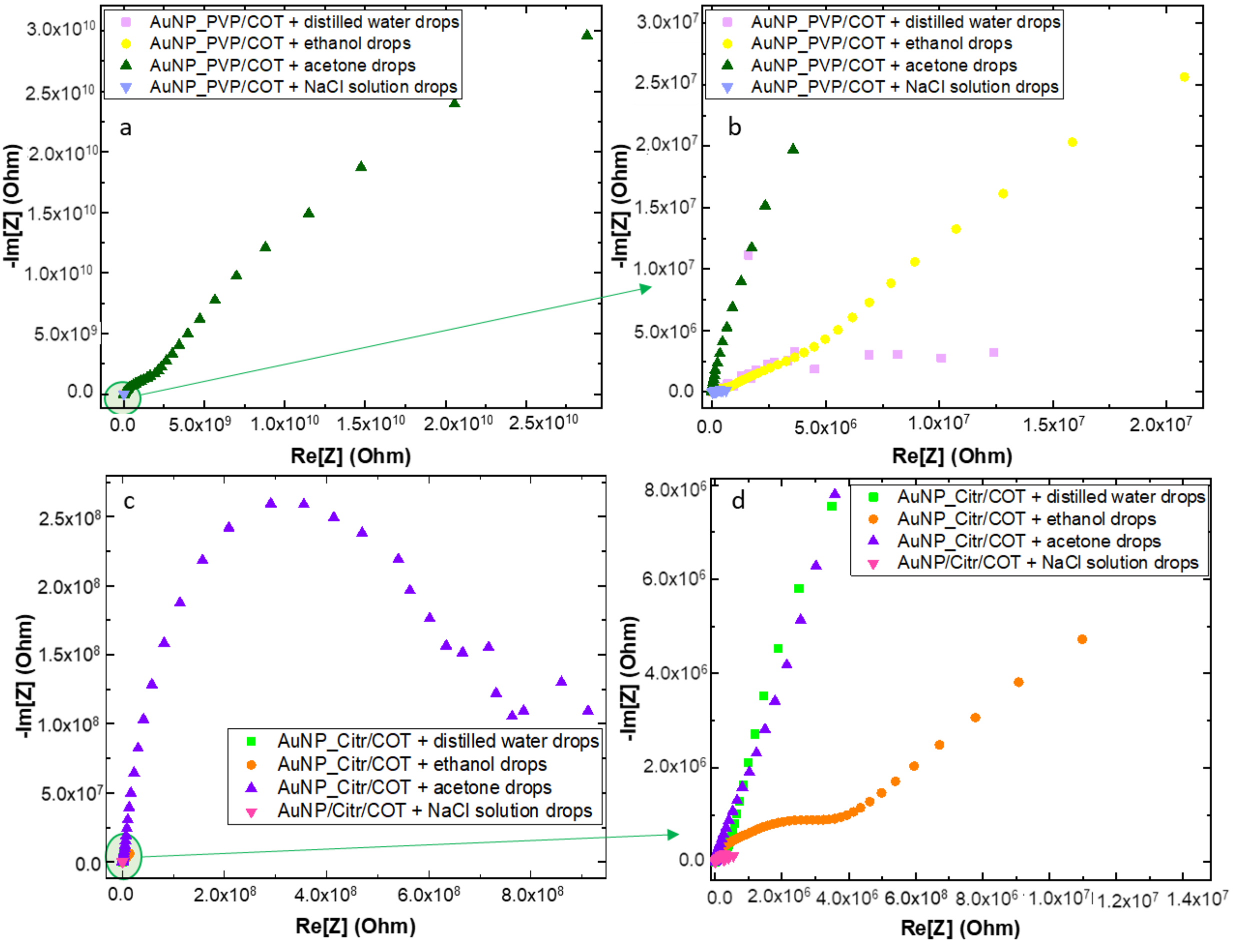


**Figure S6.** (**a**,**c**) comparison between the EIS measurements over the functionalized fabrics after spraying various probes (water, ethanol, acetone, NaCl) (**b**,**d**) zoom plot at high frequencies.

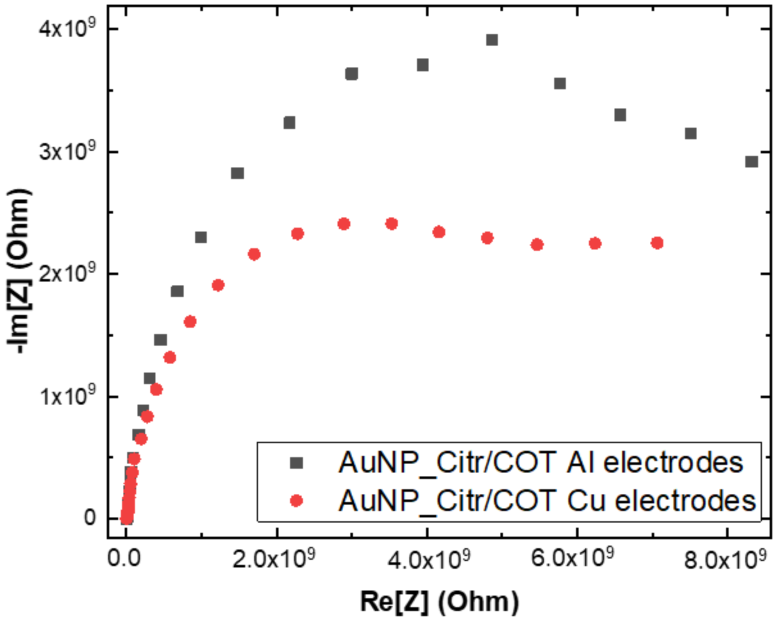


**Figure S7.** Comparison between the EIS measurements performed with Al and Cu electrodes.
